# Vehicle submersion: a review of risk factors and preventive efforts

**Published:** 2024-10

**Authors:** Zahra Asgari Tapeh, Azar Darvishpour

**Affiliations:** ^*a*^ Department of Nursing, Faculty of Nursing and Midwifery, Iran University of Medical Sciences, Tehran, Iran.; ^*b*^ Department of Nursing, Zeyinab (P.B.U.H) School of Nursing and Midwifery, Guilan University of Medical Sciences, Rasht, Iran.; ^*c*^ Social Determinants of Health Research Center, Trauma Institute, Guilan University of Medical Sciences, Rasht, Iran.

## Abstract

**Background:**

Drowning is the third leading cause of unintentional injury death worldwide, with over 300,000 fatalities annually. Vehicle submersions have a high fatality rate, yet there is limited information on this topic. Increasing awareness among vehicle occupants about risk factors and promoting quick, appropriate actions can prevent these drownings.

**Methods:**

This systematic review searched English articles in Web of Science, Google Scholar, and PubMed databases from January 2012 to July 2024 using keywords related to vehicle submersion and drowning. Qualitative evaluation was conducted using Joanna Briggs Institute tools, and data analysis was performed using qualitative content analysis.

**Results:**

Of 57 articles, 29 were included in the final review. Risk factors were categorized into two main categories: "Non-Individual Factors" with seven sub-categories (flooding, traveling on water, recreational aquatic activities, crash into open water, unguarded roads, lack of emergency rescue services, and vehicle features) and "Individual Factors" with five sub-categories (men, driving errors, use of alcohol and drug abuse, medical condition, and lack of swimming and rescue skills). Preventive efforts were grouped into four main categories: early warning with two sub-categories (dangerous roads and forecasting flood or heavy rainfall), skillful training with four sub-categories (swimming skills, escaping from a sinking vehicle, cardiopulmonary resuscitation, and checking public and local warning systems), enhancing safety with five sub-categories (personal flotation devices, effective guardrails, reflective signs and lines in roads, proper barriers, and flood risk management), and mitigating mortality with three sub-categories (medical emergency dispatch protocol, qualified healthcare providers, and appropriate equipment and facilities).

**Conclusions:**

Vehicle submersion as a significant, multifactorial, and challenging public health issue, is currently underestimated which can be attributed to insufficient data collection, inconsistent categorization, and a lack of reporting in certain regions. Establishing a framework to prevent, warn, and manage vehicle submersion incidents could reduce their frequency and lower mortality rates.

**Keywords:**

Vehicle Submersion, Drowning, Review, Aquatic accident, Water-related fatality

